# A Taxonomic Review of Attevidae (Lepidoptera: Yponomeutoidea) from China with Descriptions of Two New Species and a Revised Identity of the Ailanthus Webworm Moth, *Atteva fabriciella,* from the Asian Tropics

**DOI:** 10.1673/031.013.6601

**Published:** 2013-06-30

**Authors:** Jae-Cheon Sohn, Chun-Sheng Wu

**Affiliations:** 1 Department of Entomology, University of Maryland, 4112 Plant Sciences Building, College Park, MD 20742, USA; 2 Key Laboratory of Zoological Systematics and Evolution, Institute of Zoology, Chinese Academy of Sciences, Beijing 100101, China

**Keywords:** India, Indonesia, Malaysia, nomenclature, taxonomy, Thailand, Vietnam, Yponomeutidae

## Abstract

This review describes four species of *Atteva* (Lepidoptera: Yponomeutoidea: Attevidae) from China, including two new species: *A. wallengreni* n. sp. and *A. yanguifella* n. sp. The taxonomic identity of the Ailanthus webworm moth from South and Southeast Asia is revised with a designation of neotype *for Phalaena (Tinea) fabriciella* Swederus. Lectotypes of *Atteva brucea* Moore and *A. niviguttella* Walker are designated. *Atteva brucea* is synonymized with *A. fabriciella.* Synonymy of *Atteva niviguttella* and *A. fabriciella* is reconfirmed. The previous Chinese records of *A. fabriciella* were based on confusions with *A. wallengreni* n. sp. Confirmed specimens of *A. fabriciella* from China are reported. A pair of confused species, *A. fabriciella* and *A. wallengreni* n. sp., are distinguished by the number of white dots on the forewings and the genital features. Another confused pair, *A. niveigutta* and *A. yanguifella* n. sp., are compared by external and genital features. All type specimens of the described species are illustrated and compared with the conspecific specimens from various countries of the Asian tropics. Keys to all the species from China are provided.

## Introduction

The *Atteva* group (Lepidoptera: Yponomeutoidea) is a distinctive lineage of yponomeutoids with unique morphological features. It was once regarded as a subfamily of Yponomeutidae *sensu* Kyrki (1990), but currently it forms its own family, namely Attevidae (Heppner 1982; van Nieukerken et al. 2011). Kyrki (1984) suggested possible apomorphies for this group: i) the presence of chaetosema on head; ii) the reduced hindleg tibia and tarsus, particularly in males without tibial spurs; iii) larvae with two subventral setae on meso- and metathorax; and iv) the concealed labial palpi in the pupae. Dugdale et al. (1998) added the tegumen of male genitalia bearing an unsealed prong as another probable autapomorphy of the family. Attevids are also distinguished from other yponomeutoid groups by a combination of the characteristics, including their scape without pectens; 3-segmented maxillary palpi; lack of pterostigma; abdominal terga without spines; phallus without basal ‘scape’; W-shaped gnathos; and larvae with cranial seta V1 short and Pl not below Af2-P2 line (Dugdale et al. 1998). They often have conspicuous coloration, which is supposedly involved in a warning signal and one of the antipredator adaptations. The oligophagous larvae, if known, feed on limited numbers of plant families, such as Fabaceae, Simaroubaceae, Lauraceae, and Araliaceae, and live in communal webs loosely spun with host leaves (Dugdale et al. 1998).

*Atteva,* the only genus of Attevidae, comprises 53 species that are distributed in the Pantropics (Heppner 2009). High species diversity of *Atteva* occurs in the Indo-Australian region (ca. 35 spp.). Contrasting to such high diversity in the region, only three species of *Atteva,* i.e., *A. brucea* Moore, *A. fabriciella* (Swederus), and *A. niveigutta* Walker, have been known from China, which encompasses a vast tropical zone (Hua 2005). The low number of species currently known suggests that concentrated inventories may reveal more new species of this group from China.

The aims of this paper are to review all the known Chinese species of *Atteva,* to rectify erroneous records, to verify synonymies, and to describe two new species. The identity of the Ailanthus webworm moths, *Atteva fabriciella,* from Asian tropics, including South China, is revised and clarified, and a neotype is designated. Species comparisons and keys to distinguish all four species of *Atteva* from China are provided.

## Material and Methods

Pinned specimens from five institutional collections were examined. Besides materials from China, specimens from adjacent countries were considered for possible geographic variations.

Abbreviations for the specimen depositories are:

CAUB — Department of Entomology, China Agricultural University, Beijing, China.

IZCAS — Institute of Zoology, Chinese Academy of Sciences, Beijing, China.

BMNH — Natural History Museum (formerly British Museum of Natural History), London, UK.

OMNH — Oxford University Museum of Natural History, Oxford, UK.

USNM — United States National Museum of Natural History, Washington DC, USA.

Selected specimens were dissected following Clarke (1941) to examine genitalia and abdominal structures, except that chlorazol black was used for staining. Dissected genitalia were mounted on a slide glass, using Euparal resin (BioQuip Inc., www.bioquip.com). Slide-mounted dissections were examined under Leica MZ APO stereoscope or Leica LETTZ-DMRX microscope (www.leica-microsystems.com).

The genitalia slide numbers were given the suffix ‘IOZ’ for the specimens from IZCAS, ‘USNM’ from USNM, and ‘BMNH’ from BMNH. Unmounted genitalia and abdomens were stored in a glycerin-filled, transparent envelope attached to the corresponding pinned specimen. Terms for genitalia follow Klots (1970).

### Systematics

**Family Attevidae Genus *Atteva* Walker, 1854 A checklist of the Chinese Species of Attevidae**
*Atteva fabriciella* (Swederus, 1787)
— *brucea* Moore, 1859, **n. syn.**
= *niviguttella* Walker, 1863
*Atteva wallengreni* Sohn and Wu, **n. sp.**
*Atteva yanguifella* Sohn and Wu, **n. sp.**
*?Atteva niveigutta* Walker, 1854

**Keys to the species of *Atteva* from China based on external appearance**
**1.** Labial palpi and legs mostly dark brown2
Labial palpi and legs mostly white*A. fabriciella* or *A. wallengreni* n. sp. *
**2.** White dots on the forewings small, rarely transverse-elongate*A. yanguifella* n. sp. (Figure 13, 14)
White dots on the forewings large, often transverse-elongate*A. niveigutta* (Figure 15, 16)

* *Atteva fabriciella* with reduced white spots on the forewings is often superficially indistinguishable from *A. wallengreni* n. sp., and in such cases examination of genitalia is necessary.

**Keys to the species of *Atteva* from China based on male genitalia**
**1.** Uncus except posterolateral processes rectangular2
Uncus except posterolateral processes semicircular*A. wallengreni* n. sp. (Figure 23)
**2.** Valva elliptical3
Valva triangular*A. fabriciella* (Figure 18)
**3.** Medial gnathal plate wider apically; saccus 1/2 as long as valve*A. niveigutta* (Figure 28)
Medial gnathal plate of even width; saccus 1/3 as long as valva*A. yanguifella* n. sp. (Figure 33)

**Keys to the species of *Atteva* from China based on female genitalia**
1. Corpus bursae without apparent pleats2
Corpus bursae with pleats radiating from signum*A. fabriciella* (Figure 38)
**2.** Signum elliptical3
Signum inverted-pentagonal*A. yanguifella* n. sp. (Figure 44)
**3.** Denticules on signum scattered*A. wallengreni* n. sp. (Figure 43)
Denticules on signum aggregated near edges*A. niveigutta* (Figure 46)

**Figure 1–16.**
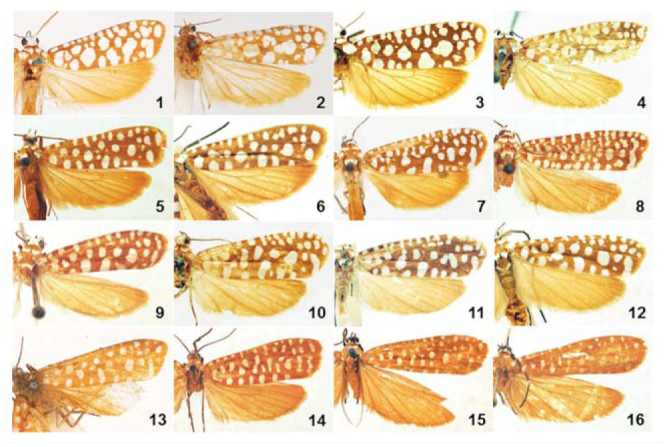
Adults of *Atteva* spp. 1–6. *A. fabriciella*. 1. Lectotype of *A. brucea*, ♂ (BMNH); 2. Lectotype of *A. niviguttella*, ♀ (BMNH); 3. Neotype, India, Coimbatore, ♀ (USNM); 4. India, Orissa, ♀ (USNM); 5. China, Yunnan, ♀ (IZCAS); 6. Thailand, Phetchabun, ♂ (BMNH). 7–12. *A. wallengreni* n. sp. 7. Holotype, China, Guangzhou, ♀ (USNM); 8. China, Hainan, ♀ (IZCAS); 9. China, Hainan, ♂ (IZCAS); 10. Vietnam, Ho Chi Minh, ♀ (USNM); 11. Malaysia, Perak, ♂ (USNM); 12. Thailand, Phetchabun, ♀ (BMNH). 13–14. *A. niveigutta*. 13. Holotype, Bangladesh, Sylhet, ♀ (BMNH); 14. India, Assam, ♀ (USNM). 15–16. *A. yanguifella* n. sp. 15. Holotype, China, Xizang, ♂ (IZCAS); 16. Paratype, China, Xizang, ♀ (IZCAS). High quality figures are available online.

**Figure 17.**
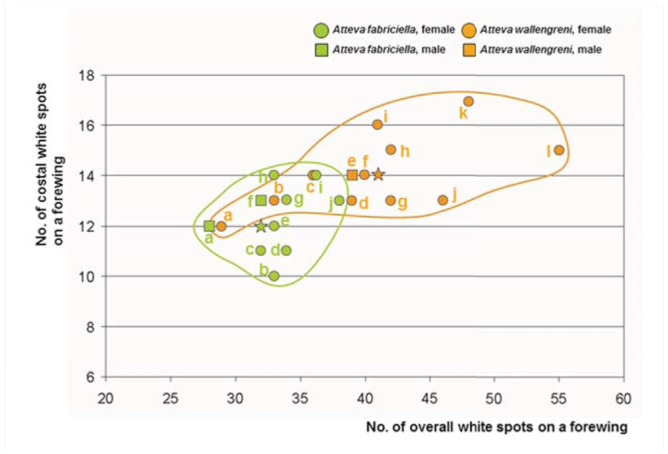
Scattergram of numbers of white dots on overall and costal area per forewing upper-side between *Atteva fabriciella* (n = 10) and *A. wallengreni* n. sp. (n = 12). Green circle = female of *A. fabriciella*; green square = male of *A. fabriciella*; orange circle = female of *A. wallengreni* n. sp.; orange square = male of *A. wallengreni* n. sp. Average numbers are shown as a green star for *A.* *fabriciella* and an orange star for *A. wallengreni* n. sp. Geographical annotations for *A. fabriciella* (a — Thailand, Phetchabun; b — China, Yunnan; c — China, Guangxi; d — Indonesia, Java; e — India, Coimbatore; f — India, Ahmabad; g — India, Coimbatore; h — India, Orissa, i — Sri Lanka; j — Indonesia, Java) and for *A. wallengreni* n. sp. (a — China, Hainan; b — China, Guangzhou; c — Vietnam, Ho Chi Minh; d — Thailand, Phetchabun; e — Malaysia, Perak; f — China, Wallengren's *fabricella* [sic]; g — China, Hongkong; h — China, Shannxi; i — China, Guangzhou; j — China, Guangzhou; k — China, Guangzhou; I — China, Guangxi). High quality figures are available online.

**Figure 18–37.**
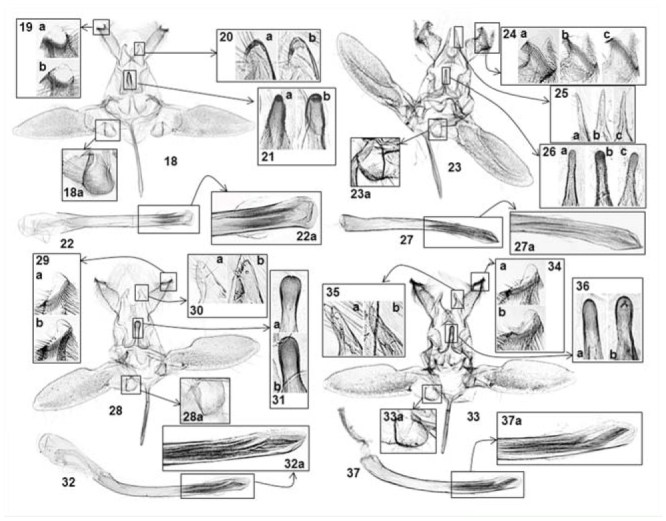
Male genitalia of *Atteva* spp. 18–22. *A. fabriciella* (a — Thailand, BMNH 32851; b — China, IOZ 09021). 18. Ventral view (18a — sacculus, close-up); 19. Comb-like setae on socius; 20. Uncal process; 21. Medial process of gnathos; 22. Phallus (22a — cornuti, close-up). 23–27. *A. wallengreni* n. sp. (a — China, IOZ 09027; b — China, IOZ 09023; China, IOZ 09026). 23. Ventral view (23a — sacculus, close-up); 24. Comb-like setae on socius; 25. Uncal process; 26. Medial process of gnathos; 27. Phallus (27a — cornuti, close-up). 28–32. *A. niveigutta* (a — India, USNM 96369; b — Thailand, BMNH 32853). 28. Ventral view (28a — sacculus, close-up); 29. Comb-like setae on socius; 30. Uncal process; 31. Medial process of gnathos; 32. Phallus (32a — cornuti, close-up). 33–37. *A. yanguifella* n. sp. (a — China, IOZ 09029; b — China, IOZ 09028). 33. Ventral view (33a — sacculus, close-up); 34. Comb-like setae on socius; 35. Uncal process; 36. Medial process of gnathos; 37. Phallus (37a — cornuti, close-up). High quality figures are available online.

**Figure 38–49.**
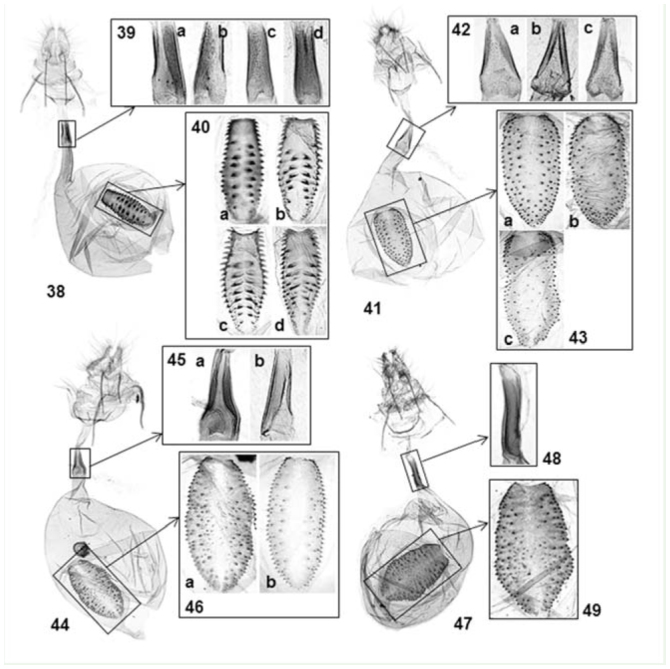
Female genitalia of *Atteva* spp. 38–40. *A. fabriciella* (a — India, USNM 96410; b — Neotype, India, USNM 96367; c — China, USNM 96375; d — China, IOZ 09022). 38. Ventral view; 39. Antrum; 40. Signum. 41–43. *A. wallengreni* n. sp. (a — Thailand, BMNH 32852; b. China, IOZ 09025; c — Vietnam, USNM 96408). 41. Ventral view; 42. Antrum; 43. Signum. 44–46. *A. niveigutta* (a — Thailand, BMNH 32854; b — India, USNM 96370). 44. Ventral view; 45. Antrum; 46. Signum. 47–49. *A. yanguifella* n. sp. (China, IOZ 85092). 47. Ventral view; 48. Antrum; 49. Signum. High quality figures are available online.

**Figure 50.**
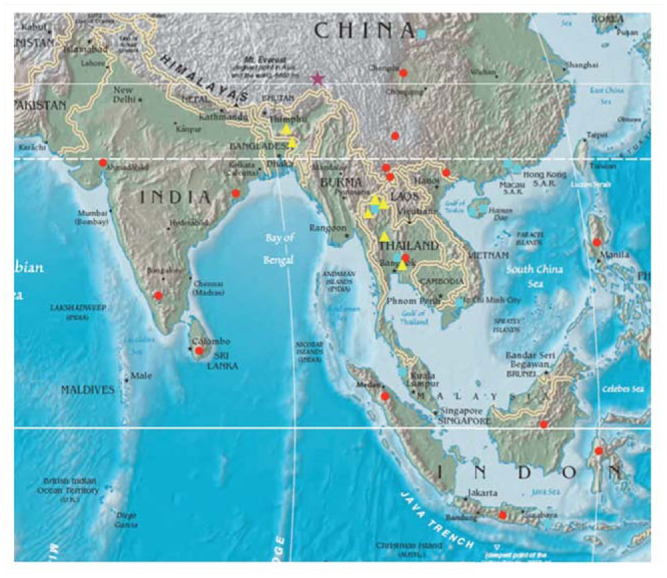
Distribution map of *Atteva* spp. Red circle = *A. fabriciella*; Blue square = *A. wallengreni*; Yellow triangle = *A.* *niveigutta*; Purple star = *A. yanguifella*. Map from www.asianews.it. High quality figures are available online.

***Atteva fabriciella* (Swederus, 1787) (Figures 1–6, 18–22, 38–40)**
*Phalaena (Tinea) fabriciella* Swederus, 1787: 277. Type locality: India.
*Atteva brucea* Moore, [1859]: 300. **n. syn.** Type locality: Indonesia.
*Corinea niviguttella* Walker, 1863: 542. Type locality: Sri Lanka. Synonymized by Walsingham and Durrant (1900: 559).
*Atteva bruceella;* Guenée, 1879: 290. An unjustified emendation of *brucea* Moore.
*Atteva niviguttella;* Guenée, 1879: 290.
*Atteva niveiguttella* [sic]; Berg, 1880: 104.
*Atteva fabriciella;* Berg, 1880: 103.
**Diagnoses**
  This species is similar to other species of *Atteva* from Asian tropics that have orange-colored forewings with white markings, especially *A. impariguttata* Zeller and *A. balanota* Meyrick. The white patches are denser in *A. fabriciella* than in *A. impariguttata.* This species is also different from *A. balanota* in the shape of the white markings on forewings: rounds or rhomboids in *A. fabriciella,* longitudinal bars in *A. balanota. Atteva fabriciella* is often indistinguishable from *A. wallengreni,* a new species described here, in external appearance. See diagnoses of *A. wallengreni* for the differences between those two species.
**Redescription**
  **Head.** Vertex and frons white; in some specimens (especially from China), a triangular, black marking on vertex. Antennae filiform, 2/3 as long as forewing costa; scape white; first 4–5 flagellomeres entirely silvery white, the resting flagellomeres gray in basal half, white in distal half. Labial palpi slender, ascending at basal 1/3, white, obtuse apically; first segment 1/2 as long as second or third segment.
**Thorax and abdomen.** Patagium and tegula white, narrowly tinged with orange basally; mesonotum orange, with a white marking posteriorly. Forelegs with coxa white; femur orange, narrowly tinged with white on interior surface; tibia lustrous, pale grayish-brown; tarsi dark grayish-brown. Midlegs with coxa white; femus orange, narrowly white ventrally; tibia grayish-brown, with a white band at base, middle, and terminal end; tarsi grayish-brown, with a white band terminally. Hindlegs with coxa white; femur to tarsi lustrous, pale yellowish-brown dorsally, white ventrally, with a white band at the end of each segment. Forewing length 11.5 to 14mm (average 13.1mm, n = 10), orange, with 28 to 38 (average 33, n = 10) white dots, some dots fusing to form bars or irregular markings; size and arrangement of dots variable, usually ones near costa and apex smaller. Hindwing orange, semitransparent interior to submarginal area. Abdomen orange, with a white band on distal end of each sternite.
**Male genitalia** (Figures 18–22). Uncus bifid, each process stout, 1/3 as long as socius; socius of almost even width, long-hairly, with a comb-like spinose, semicircular zone near to apex. Tegumen 2 × longer than socius; gnathos with digitate, spinulate, medial plate, 2/3 as long as socius. Valva elongate, triangular, densely setose except basal 1/3 of costal area; costa slightly emarginated at basal 1/3, convex medially relatively; sacculus lobate, sparsely setose. Saccus slender, 2× longer than socius. Phallus almost straight, with a spinulate zone of cornuti 1/4 as long as phallus.
**Female genitalia** (Figures 38–40). Lamellae postvaginales as a pair of shallow, semicircular sclerites, with long setae. Emargination around ostium bursae rectangular. Ductus bursae bowl-shaped near to ostium bursae, posterior 1/2 densely granulated; antrum tubular, enlarged posteriorly, with membraneous slit on a side. Corpus bursae oval, with winkles directing to a lanceolate, deticulated signum.
**Types**
  ***Phalaena (Tinea) fabriciella.* Neotype:** female, “Neotype” [a white, rectangular label with red borders and letters], “SOUTH INDIA | COIMBATORE | 6. XII. 12. | FLETCHER. COLL.”, “231”, “Genitalia slide ♀ | By J.C. Sohn | USNM 96367” [a green label]. See below for details of this designation.
***A. brucea.* Lectotype** (designated here): male, “Lectotype” [in a circular label with indigo boarders], “JAVA | HORSF[IELD]” [in a cream-colored, rectangular label], “[18]60-15 E.I.C. [= East India Company]” [in a cream-colored, rectangular label], “LECTOTYPE | *Atteva brucea* | Moore | teste J. Sohn, 2010”, BMNH; Paralectotypes: 3 females, same data as lectotype, BMNH. Moore ([1859]) described *A. brucea,* based on five specimens, four of which (all females) are still extant in BMNH.
***A. niviguttella.* Lectotype** (designated here): female, “Lectotype” [in a circular label with indigo boarders], “S. Ind[ia] | [18]61-20” [on both sides of a pale blue, circular label], “1. CORINEA NIVIGUTTELLA” [in a folded, one-line paper], “J.D.B[radley] | B.M. | Genitalia silde | No. 1673”, “LECTOTYPE | *Atteva niviguttella* | Walker | teste J. Sohn, 2010”, BMNH; Paralectotype: 1 female, Hindostan [= Coimbatore], India (J Hearsay Coll.), BMNH. Walker (1863) included 5 specimens in the type series of *Corinea niviguttella.* Of them, only two female specimens are still extant in BMNH. In the original description, collecting data of the lectotype were given as “Ceylon [= Sri Lanka]. Presented by Dr. Templeton”. Walsingham and Durrant (1900) suggested that a specimen from Ceylon here designated as lectotype is actually *A. impariguttata* Zeller, 1877. This turned out to be incorrect because JCS's type examination showed that this specimen is clearly distinct from *A. impariguttata.*
**Materials examined**
  **China. Guangxi:** 1♀, Longjin, Mt. Daqingshan, 15 May 1963 (C. Yang), CAUB. **Sichuan:** 1♀, Chengtu [= Chengdu], [no specific locality], USNM, USNM-96375. **Yunnan:** 1♂, Damenglong, Xishuangbanna (650 m a.s.l.), 10 April 1958 (YR Zhang), IZCAS; 5♂7♀, ditto, 10–13 May 1958, IZCAS; 1♂, Mengla, 15 November 1958, IZCAS; 1♂, Mengkun, 3 June 1958, IZCAS; 7♂9♀, ditto, 10 April 1982, IZCAS, IOZ-09021(♂), 09022(♀); 2♂, Pingbian, Mt. Dawei, 19 June 1956, IZCAS; 1♀, Xiaomengyang, 14 October 1957, IZCAS.
**Thailand. Phetchabun:** 1♂, Khao Khejo, Khao Yai National Park (1200 m a.s.l.), 28 February–1 March 1989 (AM Cotton and IJ Kitching), BMNH, BMNH-32851.
**India. Orissa:** 1♀, Bhubaneshwar (500 ft), 14 February 1975 (ML Ripley), USNM, USNM-96410. **Gujarat:** 1♂, Ahmedabad, “C. No.: 1648” (Fletcher), reared from *Ailanthus* leaves, USNM.
**Distribution** (Figure 50)
  China (Guangxi; Sichuan; Yunnan), Thailand, Indonesia (Borneo; Java; Sulawesi; Sumatra), Philippines (Luzon), India, and Sri Lanka. Walker (1854) designated a syntype of *C. niviguttella* from Australia. Turner (1898) cited this record. Walsingham and Durrant (1900) suggested that this syntype could be a distinct species. This specimen no longer exists in the type series of *C. niviguttella.* It is very likely that the specimen was confused with *A. niphocosma* Turner 1903 or *A. megalastra* Meyrick 1907. In fact, *A. fabriciella* (or its synonym, *A. brucea*) has never been recorded again from Australia (Edwards 1996) since then.
**Host plant**
  **Simaroubaceae.** *Ailanthus excelsa* Roxb. (Fletcher 1914; Mishra and Pandey 1966); *Ailanthus triphysa* (Dennst.) Alston. (Verma 1992); *Brucea sumatrana* Roxb. (Horsfield's record cited in Moore [1859]); *Quassia indica* (Gaertn.) Nooteboom (Mohanadas and Verma 1984). Host records of *A. fabriciella* from *Boswellia serrata* Triana et Planch. (Burseraceae) and *Santalum album* L. (Santalaceae) by Beeson (1941) and Browne (1968) need further verification. Robinson et al. (1994) mentioned that some specimens of this species from India in the BMNH collection have rearing records from *Acacia* (Leguminosae). This seems to be due to misidentification of host plants, since no additional records of the larvae feeding on *Acacia* have been made to date.
**Neotype designation of *Phalaena (Tinea) fabriciella***
  Swederus (1787) described *Phalaena (Tinea) fabriciella,* currently *A. fabriciella,* from India. The original description of the species was very short and did not include any illustration. This inadequate description can apparently be applied to more than one valid species, making the true identity of *Phalaena (Tinea) fabriciella* dubious. Wallengren (1861) redescribed *Phalaena (Tinea) fabriciella* with misspelling in specific name, i.e. *fabricella* [sic]. Wallengren's redescription included an illustration of *fabriciella,* based on a specimen from China, not from the original type locality, India. This has caused substantial confusion about the identity of *A. fabriciella* and obscured the actual distribution and host range of the species.
  Berg (1880) treated the specimen described by Wallengren as *Phalaena (Tinea) fabriciella* and stated the type locality of the species as China. Probably from this, Wallengren's specimen has been erroneously regarded as the type specimen of *Phalaena (Tinea) fabriciella* from the database of the Swedish Museum of Natural History (www2.nrm.se/en/lep_nrm/f/phalaena_fabriciella.html). Berg's view was also followed by Robinson et al. (1994), who provided a photo of *A. fabriciella* that was the same as what Wallengren (1861) illustrated. In a different study, Walsingham and Durrant (1900) suggested that Swederus and Wallengren described two different species of *Atteva,* followed by Meyrick (1914). The type specimen of *Phalaena (Tinea) fabriciella* has not been found or traced yet. Therefore, it is hard to prove which of those two views is right.
  Swederus (1787) stated that the specimen was from Mr. J. Lee among the several collectors. Considering a letter sent by F. W. Hope to J. O. Westwood (see Smith 1986: 11 and 133), the Lee collection had most likely been housed in the OUMN. The first author (JCS) searched for this specimen at the museum and also at other major European collections, but could not find it. In fact, none of lepidopteran type specimens described by Swederus have been found (see Poole 1989). It is very likely that the type specimens of *fabriciella* have been lost. To make the identity of *Phalaena (Tinea) fabriciella* clear, designation of a neotype is necessary.
  The name, *A. fabriciella,* also known as the Ailanthus webworm moth from India, has appeared in the voluminous forestry literature (e.g., Beeson 1941; Browne 1968; Mathur et al. 1970; Singh and Sharma 1979; Mohanadas and Verma 1984; Verma 1992; Nair 2007). Especially, Fletcher (1914: 462) and Mishra and Pandey (1966: 465) illustrated *A. fabriciella.* Those illustrations showed that the species is apparently different from Wallengren' s *fabriciella* and conspecific with *A. brucea.* Examination of *Atteva* specimens from various regions of the Asian tropics revealed that Wallengren' s *fabriciella* does not occur in India. This contradicts Berg (1880). Swederus (1787) stated that *Phalaena (Tinea) fabriciella* has more than 30 white dots on the forewings. Wallengren' s *fabriciella* (= *Atteva wallengreni* n. sp.) frequently has more than 40 dots (Figure 17). *A. fabriciella* illustrated by Fletcher (1914) and Mishra and Pandey (1966) have 35 dots and 32 dots respectively. This indicates that the specimens of *A. fabriciella* stated from the Indian forestry literatures better fit with the original description of *A. fabriciella.* To clarify the identity of *Phalaena (Tinea) fabriciella,* it is proposed to designate as neotype a female specimen from Coimbatore, India. This specimen is consistent with the original description in the locality and characteristics as recommended by the ICZN (Article 75.3.5 and 75.3.6). This designation also meets the Article 75.6 of the Code, conserving the prevailing usage of *Phalaena (Tinea) fabriciella* in the voluminous Indian literature.
**Synonymic notes**
  *A. brucea* is here proposed to be a junior synonym of *A. fabriciella* according to designation of a neotype for *Phalaena (Tinea) fabriciella.* The female genitalia of *A. niviguttella* from the type series was examined, and it was found to be identical with *A. brucea* (= *A. fabriciella*), as already suggested by Walsingham and Durrant (1900). Becker (2009) stated that *A. niveigutta* is a synonym of *A. fabriciella.* This seems to be due to a confusion of the two similar names, *niveigutta* and *niviguttella. Atteva niveigutta* and *A. fabriciella* are two separate species, and are easily distinguished by external appearance.
**Faunistic notes**
  *A. fabriciella* was first recorded in China by Wallengren (1861), followed by Meyrick (1914), Fletcher (1929) and Wu (1938). This record, however, turned out to be due to a misidentification. Wallengren's (1861) specimen of *fabriciella* is in fact a distinct species from *A. fabriciella.* Therefore, the specimens reported from our study are actually the first record of *A. fabriciella* from China.

***Atteva wallengreni* n. sp. (Figure 7–12, 23–27, 41–43)**
*Amblothridia fabricella* [sic!]: Wallengren 1861: 385.
**Diagnoses**
  This new species has long been confused as *A. fabriciella.* Both species are so similar that they cannot be securely separated by external appearance alone. *A. wallengreni* usually have more white dots on the forewings (frequently > 40) than *A. fabriciella* (rarely > 40). When fused, those dots appear transverse-elongate in *A. wallengreni,* whereas rhomboids or larger dots in *A. fabriciella.* For reliable identification, examination of genitalia is necessary for both species as it reveals unambiguous differences: a comb-like spinose area on socii and a pair of processes on the top of uncus larger and more slender respectively in *A. wallengreni* than in *A. fabriciella;* costa of valva without protrusion in *A. wallengreni;* corpus bursae with no apparent pleats in *A. wallengreni;* signum broader and denticules on signum smaller in *A. wallengreni* than in *A. fabriciella.*
**Description**
  **Head.** Vertex and frons white; usually a black, triangular marking between antennal scapes (absent in some individuals). Antennae filiform, 1/2 as long as forewing costa; scape white; flagellum dark brown, first 4–5 flagellomeres silvery-white dorsally. Labial palpi slender, upcurved, obtuse apically, white, intermixed with dark brown; first segment 1/2 as long as second or third segment.
**Thorax and abdomen.** Patagium orange on basal half, white on distal half; tegula orange, with a white marking outer-posteriorly; mesonotum orange, with a white band near to mesoscutellum. Forelegs with coxa white; femur orange; tibia to tarsi dark grayish-brown. Mid- and hindlegs with coxa white; femur to tarsi lustrous, pale grayish-brown dorsally, white ventrally, with a white ring at the end of each segment. Forewing length 11 to 15 mm (average 14.3mm, n=12), elongate, orange, with 29 to 55 (average 41, n=12) white dots, some (especially submarginal dots) fused to form bar-like marking. Hindwing orange, semitransparent near to base. Abdomen orange, with a white band on distal end of each sternite.
**Male genitalia** (Figure 23–27). Uncus trapezoidal, emarginated posteriorly, with a pair of slender processes as long as socius; socius rectangular, long-hairy ventroposteriorly, with densely setose, triangular extension apically and a straight, comb-like spinose zone subapically. Tegumen as long as socius; gnathos with slender, spinulate medial plate. Valva elongate, elliptical, densely setose except costal base; costa slightly emarginated at basal 1/3; sacculus slightly swollen at base. Saccus slender, 1/2 as long as valva. Phallus almost straight, with cornuti comprising various size of spinules in terminal half.
**Female genitalia** (Figures 41–43). Lamellae postvaginales as a pair of sclerotized, semicircular, setose lobes. Apophyses anteriores broader than posteriores. Ductus bursae as long as corpus bursae, shallow bowl-shaped near to ostium bursae, with antrum at middle; antrum long-conical, with membraneous slit on a side and triangular opening anteriorly. Corpus bursae globular; signum as an elliptical plate, with sparse, small denticules.
**Types**
  **Holotype.** Female, Canton, Guangzhou, Guangdong, China, September 1920, CW Howard, Collector, USNM.
**Paratypes** — 2♀, same data as holotype, USNM, USNM-96409.
**Materials examined**
  **China. Shaanxi:** 1♀, Ningshan Co., Huoditang (1580–1650 m a.s.l.), 30 June 1999 (D. Yuan), IZCAS. **Guangdong:** 1♀, Guangzhou, Shipai, 8 October 1957 (Guan), CAUB. **Hainan:** 1♀, Koreisi, no date 1972 (Issiki), USNM, USNM-96422; 3♂, Wangning (60 m a.s.l.), 4–6 March 1963 (BL Zhang), IZCAS; 3♂2♀, ditto, 12–14 March 1963, IZCAS, IOZ-09024(♀); 6♂5♀, no. 271,no specific locality, 23 June 1958, IZCAS, IOZ-09026(♂); 1♂, Bawangling (N 19° 1′, E 109° 1′, 145 m a.s.l.), 7 May 2007, IZCAS, IOZ-09023; 2♀, Jianfengling, 8 May 1978, IZCAS; 1♀, Sarna, 12 May 1936 (G Ros), IZCAS, IOZ-09025; 1♂3♀, Xinglong, April 1963 (BL Zhang), IZCAS, IOZ-09027(♂). **Hongkong:** 5♀, Sek Kwu Chau, 3 July 1996 (G Reels), BMNH.
**Vietnam. Hochiminh:** 1♀, Saigon (= Ho Chi Minh City), no collecting date, USNM, USNM-96408.
**Thailand. Chiang Mai:** 2♀, Doi Suthep-Pui National Park (1460 m a.s.l.), 26 April–10 May 1989 (AM Cotton), BMNH; 1♀, ditto (1490 m a.s.l.), 19 September 1990 (IJ Kitching and AM Cotton), BMNH. **Phetchabun:** 1♀, Khao Khejo, Khao Yai National Park (1140 m a.s.l.), 1 September 1986 (GS Robinson), BMNH, BMNH-32852.
**Indonesia. Bali:** 1♀, Candi Dasa, 7 VI 1985 (RM Pearsoa), at light, BMNH.
**Malaysia. Perak:** 1♂, Taipin [= Taiping], 14 IX 1911 (T Shiraki), USNM, USNM-96376.
**Distribution** (Figure 50)
  China (Guangdong; Hainan; Hongkong; Shaanxi), Indonesia (Bali), Malaysia (Perak, new record), Thailand and Vietnam (Hochiminh, new record).
**Etymology**
  This species is named after Hans Daniel Johan Wallengren (1823–1894), the Swedish entomologist who described this species under the name *Amblothridia fabricella* [sic].
**Synonymic notes**
  This species has long been confused with *A. fabriciella.* The confusion stemmed from Wallengren (1861), who redescribed the latter species with a specimen from China. Walsingham and Durrant (1900) suggested Wallengren' s *fabriciella* was conspecific with *A. niveigutta.* Later, Meyrick (1914) listed it as a synonym of *A. brucea.* In fact, the species illustrated by Wallengren (1861) is not consistent with *A. brucea* (= *A. fabriciella*) or *A. niveigutta* in collecting locality and morphological features (see keys in this paper).

***Atteva niveigutta* Walker 1854 (Figure 13–14, 28–32, 44–46)**
*Atteva niveigutta* Walker, 1854: 526; Meyrick 1914: 22; Wu 1938: 377.
*Atteva niveiguttata* [sic]; Berg, 1880: 103.
**Diagnoses**
  This species is similar to *Atteva aleatrix* Meyrick, described from Fiji Island, but differs from the latter by having denser white dots on the forewings. It is also easily distinguished from *A. fabriciella* and *A. wallengreni* n. sp. by the labial palpi and the legs, which are mostly dark brown (white in the latter two).
**Description**
  **Head.** Vertex white with a triangular, black marking; frons black, tinged with white laterally. Antennae filiform, 1/2 as long as forewing costa; scape white in basal half, dark brown in distal half; flagellum dark brown, first 3–4 flagellomeres with lustrous dark gray scale-covering dorsally on basal half. Labial palpi slender, obtuse apically, ascending after basal 1/4; first segment white, 1/2 as long as second or third segment; second and third segments dark brown, sparsely intermixed with white.
**Thorax and abdomen.** Patagium orange with narrowly white posteriorly; tegula orange; mesonotum orange, with a white band near to mesoscutellum. Forelegs with coxa white; femur orange, narrowly paler on interior surface; tibia to tarsi dark grayish-brown. Mid- and hindlegs with coxa white; femur dark brown dorsally, white ventrally; tibia dark brown a white band at both ends and middle; tarsomeres dark brown, with a white band terminally. Forewing length 13 to 16mm (average 15mm, n = 4), elongate, lustrous, orange, with about 27 to 53 (average 45, n = 4) white dots, some fused to form dashes especially on distal and dorsal areas, dots near costa smaller. Hindwing orange. Abdomen orange dorsally, fuscous orange ventrally with a white band on distal end of each sternite.
**Male genitalia** (Figure 28–32). Uncus bifid, each process stout, 1/3 as long as socius; socius narrower to apex, with a comb-like spinose zone subapically. Tegumen as long as socius; gnathos with a densely ciliate, digitate medial plate, apex slightly broadened. Valva elongate, elliptical, slightly emarginated at basal 1/3 of costa, densely setose except on basal area; sacculus lobate at base, setose. Saccus slender, 1/2 as long as valva. Phallus slightly bent at basal 1/5; cornuti comprising a minute spinules of zone 1/2 as long as phallus and 5 to 6 stout spines terminally.
**Female genitalia** (Figure 44–46). Lamellae postvaginales as a pair of shallow, setose, semielliptical lobes. Emargination near to ostium bursae rectangular. Ductus bursae 1/2 as long as corpus bursae, bowl-shaped near to ostium, with antrum at anterior 1/3; antrum tubular, slightly enlarged to anterior, with triangular slit anteriorly. Corpus bursae oval; signum as a narrow, elliptical plate, weakly sclerotized medially, with denticules marginally or submarginally.
**Types**
  **Holotype.** Female, holotype on a circular label with red borders, East India (Bangladesh, Sylhet region) on a pale blue label, J.D.Bradley, B.M. Genitalia slide No. 1671, 1. Atteva niveigutta on a single line paper, BMNH.
**Materials examined**
  **India. Assam:** 1♀, Khasi Hills, no collecting date, collected by W. Schaus, USNM, genitalia slide no. USNM-96370; 1♂, Khasias, Cherra Punji, 1894 (Swinhoe), USNM, genitalia slide no. USNM-96369.
**Thailand Mae Chaem distr.:** 1♂, 1♀, Doi Inthanon National Park, Checkpoint 37km (alt. 1700 m a.s.l.), 25–27 August 1987 (MG Allen), BMNH; 6♂, 4♀, ditto, 9–10 September 1988, (MG Allen, AM Cotton, and IJ Kitching), at light, BMNH. **Nan:** 1♂, 4♀, Doi Phu Kha National Park, 35.4 km (1540 m a.s.l.), 28 July 1990 (IJ Kitching and AM Cotton), BMNH, BMNH-32853(♂), 32854(♀). **Chiang Mai:** 1♂, Doi Suthep-Pui National Park (1200 m a.s.l.), 5 May 1988 (GS Robinson), BMNH; 1♀, ditto (1250 m a.s.l.), 29 May 1989 (IJ Kitching and AM Cotton), BMNH; 1♂, ditto (1440 m a.s.l.), 29 April–4 May 1988 (GS Robinson), BMNH; 1♂, ditto (1460 m a.s.l.), 7 March 1989 (AM Cotton and IJ Kitching), BMNH; 1♂, ditto, 26 April– 10 May 1989 (AM Cotton), BMNH; 1♂, 1♀, Chiang Dao, San Pakia RFD, Watershed Station (1450 m a.s.l.), 28 April–1 May 1994 (IJ Kitching et al.), BMNH. **Uthai Thani:** 1♂, Huai Kha Khaeng Wildlife Sanctuary, Khao Nang Ham viewpoint (500 m a.s.l.), 30–31 October 1991 (IJ Kitching and AM Cotton), BMNH **Nakhon Ratchasima:** 1♂, Khao Yai National Park (800 m a.s.l.), 1 September 1988 (MG Allen), BMNH; 1♂, ditto (1200 m a.s.l.), 7 February 1986 (MG Allen), BMNH; 1♂, ditto, 19 April 1988 (TW Harman), BMNH.
**Host plant**
  **Simaroubaceae.** *Ailanthus excelsus* Roxb. (Moore 1867).
**Distribution** (Figure 50)
  Bangladesh, China (?), India (Assam), and Thailand.
**Faunistic notes**
  Caradja (1926) first listed this species for the Chinese fauna. Since then, no additional records have been made. Given the prevalent confusion of *niveigutta* with *fabricella* sensu Swederus or Wallengren, it is probable that Caradja misidentified it with one of those or the new species described next.

***Atteva yanguifella* n. sp. (Figure 15–16, 33–37, 47–49)**
**Diagnoses**
  This new species is very similar to *A. niveigutta,* but is distinguished from the latter by having rather reduced white dots on the forewings. The two species also differ in male and female genitalia (Table. 1).
**Description**
  **Head.** Vertex dark brown, surrounded with white marginally; fions white. Antennae filiform, 1/2 as long as forewing; scape white on basal half, dark brown on distal half; flagellomeres dark gray. Labial palpi slender, upcurved; first segment as long as antennal scape, white; second segment 2× longer than first; third segment as long as second; second and third segment dark brown, sparsely intermixed with white.
**Thorax and abdomen.** Patagium orange, tinged with white posteriorly; tegula orange, with white marking posteroventrally; mesonotum orange, edged with white along scutoscutellar suture. Foreleg with coxa dark brown, intermixed with orange on lower surface, white on upper surface; femur dark brown dorsally, white ventrally; tibia dark brown, with white marking on distal 1/3; tarsi dark brownish-gray. Midleg with coxa orange, tinged with orange-white terminally; femur dark brown ventrally orange-white dorsally; tibia dark brownish-gray, with a white band on the middle and the distal end; tarsi dark brownish-gray; first tarsomere with a white ring on the distal end. Hindleg with coxa orange, mottled with white; femur dark brown, tinged to orange distally; tibia with dense hair tufts orange dorsally, yellowish-white ventrally; tarsi very slender, with gradual change from orange on base to white-orange to dark gray on distal end. Forewing 13.5 to 17.5mm (average 15.3mm, n = 3), elongate, with obtuse apex and straight termen, orange to orange-brown; 25 to 35 (average 30, n = 3) white markings scattered throughout the forewing. Hindwing as narrow as forewing, with broadly round termen, orange. Abdomen orange, with white marking on distal end of each sternite.
**Male genitalia** (Figure 33–37). Uncus rectangular, bifid, each process 1/2 as long as socius; socius narrower to apex, long-hairy, with a comb-like spinose zone near to apex; Tegumen as long as socius; gnathos starting from broad arm and then curved perpendicularly at 1/3 and 2/3 respectively, with a densely ciliate, digitate medial plate. Valva elongate, elliptical, 2.5× longer than socius, densely setose except on basal area; costa slightly emarginated at basal 1/3 and convex at middle; sacculus lobate, setose at base. Saccus slender, 1/3 as long as valva. Phallus slightly bent at basal 1/4; a spinulate zone of cornuti 2/5 as long as phallus, with 4 to 5 of stout spines terminally.
**Female genitalia** (Figure 47–49). Lamellae postvaginales as a pair of shallow, semicircular, setose lobes. Apophyses posteriores as long as anteriores. Ostium bursae surrounded by semicircular emargination. Ductus bursae bowl-shaped near to ostium bursae, with antrum at anterior 1/4; antrum cylindrical, with membraneous slit on a side. Corpus bursae oval, 1.4× longer than ductus bursae, with protruding cervical area; signum elongate, inverted pentagonal, narrowly less sclerotized medially, with denticules denser to margin.
**Types**
  **Holotype.** Male, Xizang Motuo [=Medog], alt. 850 m, 30 May 1983 [in Chinese], IZCAS, IOZ-09028.
**Paratypes.** 3♂1♀, same as holotype for locality and collecting date, IZCAS, IOZ-09029; 1♀, same locality as holotype, 24 March 1983 (YH Han), IZCAS, IOZ-85092.
**Distribution** (Figure 50)
  China (endemic: Xizang).
**Etymology**
  The species epithet is derived from ‘Yanguifei,’ one of the four legendary beauties in Chinese history, and refers to the beautiful coloration of the new species.

**Table 1.**
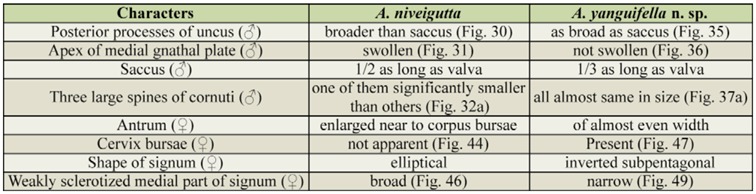
Comparison of the genital features between *Atteva niveigutta* and *A. yanguifella* n. sp.

## Abbreviations

**BMNH**,: Natural History Museum
**CAUB**,: China Agricultural University, Beijing
**IZCAS**,: Institute of Zoology, Chinese Academy of Sciences
**OMNH**,: Oxford University Museum of Natural History
**USNM**,: United States National Museum of Natural History
